# Isolation of endophytic bacteria from the leaves of *Anredera cordifolia* CIX1 for metabolites and their biological activities

**DOI:** 10.1186/s12906-020-03095-z

**Published:** 2020-10-07

**Authors:** Celiwe Innocentia Nxumalo, Londeka Sibusisiwe Ngidi, Jabulani Siyabonga Emmanuel Shandu, Tsolanku Sidney Maliehe

**Affiliations:** Faculty of Science and Agriculture, Department of Biochemistry and Microbiology, University of Zululand, Private Bag X1001, KwaDlangezwa, KwaZulu Natal Province 3886 South Africa

**Keywords:** *Pseudomonas aeruginosa* CP043328.1, Antibacterial activity, Antioxidant activity, Volatile compounds

## Abstract

**Background:**

Endophytes, especially those that are found from ethnopharmacologically noteworthy medicinal plants have attracted attention due to their diverse bioactive metabolites of pharmacological importance.

**Methods:**

This study aimed at isolating endophytic bacterium from the leaves of *Anredera cordifolia* CIX1 for its bioactive metabolites. The endophytic isolates were identified by 16S rRNA sequence and investigated for antibiotic sensitivity using different antibiotics. The secondary metabolites were evaluated for antibacterial activity against four bacterial strains. The 2-diphenyl-1-picrylhydrazyl (DPPH) and 2, 2′-azinobis (3- ethylbenzothiazoline-6-sulfonic acid) (ABTS) methods were used to assess their scavenging activities. The chemical components were analysed by gas chromatography-mass spectrometry (GC-MS).

**Results:**

Out of 13 isolates, Isolate 1 was identified as *Pseudomonas aeruginosa* CP043328.1. It was resistant to clindamycin, ertapenem, penicillin G, amoxicillin, cephalothin and kanamycin but sensitive to imipenem, meropenem, and gentamycin. Its extract demonstrated antibacterial activity with minimum inhibitory concentration value of 0.098 against *Bacillus cereus* (ATCC 10102) and *Staphylococcus aureus* (ATCC 25925) and 0.391 mg/ml against *Escherichia coli* (ATCC 25922) and *Proteus mirabilis* (ATCC 25933). The extract revealed DPPH and ABTS scavenging activities with half maximal inhibitory concentration value of 0.650 mg/ml and 0.15 mg/ml, respectively. The GC-MS revealed a total of 15 compounds with diisooctyl phthalate (50.51%) and [1, 2, 4] oxadiazole, 5-benzyl-3 (10.44%) as major components.

**Conclusions:**

*P. aeruginosa* CP043328.1 produced secondary metabolites with antibacterial and antioxidant activities.

## Background

South Africa has remarkable successes in treatment of infectious diseases that include HIV and TB, malaria and others. This is evidenced by the increase in life expectancy over the years. The increase stems from the implementation of some health initiatives that include the use of antiretroviral agents for people living with HIV [1]. Although an outstanding progress has been made in reducing morbidity and mortality rates due to infectious diseases, new microbial resistance mechanisms threaten prevention and treatment procedures [2]. This is due mainly to inadequate dosing, poor quality drugs, and genetic plasticity of the microorganisms [3]. Without effective antimicrobial agents, the success of even minor surgeries can be of a great challenge [4]. Therefore, joint efforts to discover and develop new drugs is imperial.

Medicinal plants are the predominant sources for bioactive compounds used in modern-day drug discoveries and developments [5]. Over 80% of drugs on the market shelves currently have their origin in medicinal plants [6]. Although plant metabolites exhibit strong physiological activities, their production are not of convenience in terms of quality and productivity due to hysterical environmental conditions and rapid climatic changes [7]. Thus, bioprospecting of bioactive metabolites from microorganisms have become a promising alternative route for drug discovery [8].

Microorganisms such as bacteria and fungi can produce high quality metabolites on a large scale under optimized culture conditions [9]. This is because their metabolite production is reproducible, weather and season independent [10]. Microorganisms often produce metabolites that display distinctive molecular skeletons which are not even available in the chemical libraries [11]. Endophytes are microorganisms which inhabit within plant tissues and often occur as symbionts [12]. Endophytes, especially those that are found from ethnopharmacologically noteworthy medicinal plants have attracted attention due to their diverse bioactive metabolites against pathogens [13]. They produce a plethora of metabolites with unique structures and profound biological activities. These include essential enzymes, alkaloids, flavonoids, phenolic acids, quinines, steroids and terpenoids. Although these metabolites were primarily beneficially to the host plant, they have found a wide-ranging application industrially. They are harvested and used as agrochemicals, antibiotics, immunosuppressants and antioxidants [14]. Nevertheless, most of research focuses on fungal-based bioactive products and a limited number has been isolated from bacterial endophyte [15].

*Anredera cordifolia* is a well-recognized medicinal plant that belong to Basellaceae family. *A. cordifolia* possesses pharmacological activities such as antibacterial [16], gastroprotective [17], antivirus [18], antidiabetic [19], anti-inflammatory [20], wound healing [21] and antioxidant [22]. Phytochemical screening has revealed the presence of flavanols [18], glycosides, alkaloids [23], flavonoids [24], saponin and steroid [19] in different parts. Although there are many studies documenting its medicinal properties and usage, to the best of our knowledge, there are no studies reporting on its bacterial endophytes.

The study was designed to isolate and identify endophytic bacteria from *A. cordifolia*. The bacteria were screened for extracellular enzyme and antimicrobial agent production. Moreover, its biological activities such as antibacterial and antioxidant activities were evaluated from the extracted secondary metabolites of one of the isolates. Lastly, metabolites composition was analysed. The other isolated bacteria will be investigated in future studies.

## Methods

### Chemicals and media

All chemicals and media used were procured from Sigma-Aldrich and Merck (Pty) Ltd. The water used was distilled and autoclaved.

### Sample collection and treatment

Healthy fresh leaves of *Anredera cordifolia* were collected on the 2nd of July 2019 from KwaDlangezwa area in the city of Umhlathuze, KwaZulu-Natal Province, South Africa (28 °45 ′S31 °54 ′E). The voucher specimen for *A. cordifolia* species, voucher number CIX1, was prepared and deposited in the University of Zululand Herbarium [ZULU], which is available mainly to researchers. The leaves were washed thoroughly under running tap water and air dried. The surfaces of the leaves were disinfected by dipping them in 75% ethanol for 5 min, followed by dipping in sodium hypochlorite (2% w/v NaOCl) for 4 min [10]. They were further dipped in sodium bicarbonate (10% NaHCO3) for 2 min and subsequently rinsed in sterile distilled water six times. The excess water was dried under laminar airflow chamber. To confirm the disinfection process, aliquots of the sterile water used in the final rinse were plated onto nutrient agar (NA). The plates were then incubated at 28 °C for 5 days and observed for any microbial growth [25].

### Isolation of endophytic bacteria

NA was used for the isolation of the endophytic bacteria. The disinfected leaves, without midribs, were ground in sterile 6 ml of aqueous saline solution (0.9% NaCl) using a sterile mortar, under aseptic conditions. The tissue extract was serially diluted in sterile aqueous solution. About 100 μl of each dilute (10^− 1^ and 10^− 2^) and undiluted sample were pipetted on NA plates and spread evenly using a sterilized glass spreader. Plates were sealed and incubated at 28 °C for 5 days. The colonies were counted as colony forming units (CFU) per gram and expressed as population density. The colonies were selected based on divergence in morphology, size and colour. They were then sub-cultured twice on NA and stored at 4 °C [16].

### Screening for enzyme production

#### Amylase production

The amylase production was investigated by starch agar plate method. Briefly, NA medium was supplemented with 1 g/L of starch. One microliter of the isolates was inoculated onto the medium and incubated at 28 °C for 5 days. The positive controls were *Escherichia coli* (ATCC 25922) and *Bacillus cereus* (ATCC 10102) while autoclaved distilled water served as a negative control. The plates were then flooded with Gram’s dye solution. The presence of halos around the colonies were indicative amylase production [26].

#### Proteinase production

Proteolytic production of the bacterial strains was assessed on protease casein medium that contained NA supplemented with 1% casein. One microliter of each isolate was pipetted on the plate and incubated at 28 °C for 5 days. The positive control was *Bacillus cereus* (ATCC 10102) while autoclaved distilled water served as a negative control. After incubation time, the clear zones due to casein hydrolysis were considered as an indication of protease production [27].

#### Cellulase production

Carboxy methyl cellulase production was evaluated using the method by Zamani et al. [28]. The NA supplemented with 1% of carboxy methyl cellulose was inoculated with the isolates and incubated for 5 days at 28 °C. The negative control was *Escherichia coli* (ATCC 25922) while *Cellulomonas humilata* ATCC 2517 served as the positive control. The plates were then flooded with a solution of 0.5% Congo red. Isolates forming yellow coloured zones were considered positive for cellulase activity.

#### Esterase production

The medium containing peptone 10.0 g/L, NaCl 5.0 g/L, CaCl2.2H2O 0.1 g/L, agar 18.0 g/L, pH 7.0 was prepared for determining esterase activity. To the sterilized culture medium, previously sterilized Tween 80 (1% (v/v) was added. Endophytic bacterial isolates, *E. coli* (ATCC 25922) (negative control) and *Bacillus cereus* (ATCC 10102) (positive control) were separately pipetted onto the medium and incubated at 28 °C for 5 days. After incubation period, the appearance of a well visible halo around the colonies was indicative of the esterase production [29].

#### Screening for antimicrobial agents production

Pure endophytic bacterial isolates were pre-cultured overnight on NA at 37 °C. A loopful of fresh endophytic bacterial species were inoculated into 100 ml Erlenmeyer flask containing 50 ml of sterilised nutrient broth (NB). They were incubated at 28 °C, 130 rpm for 72 h. Two millilitres of the fermented broth were centrifuged at 13000 rpm for 15 min. The culture supernatant was used for screening of antibacterial activity. An agar well diffusion method was employed to screen antibacterial activity of the isolates in accordance to [30]. Briefly, test bacterial inoculums *Bacillus cereus* (ATCC 10102), *Staphylococcus aureus* (ATCC 25925), *Proteus mirabilis* (ATCC 25933) and *Escherichia coli* (ATCC 25922) were adjusted to 106 CFU/ml. The test bacterial strains were streaked on Mueller Hinton (MH) agar with sterile swabs over the agar surface. About 6 mm diameter wells were made on the bacterial lawn using sterile cork borer. A total of 100 μl of the centrifuged endophyte broths-supernatants was pipetted into the wells. MH broth was set as negative control and ciprofloxacin (20 μg/ml) as a positive control. The plates were left at room temperature for 10 min to allow the diffusion of the supernatants into the agar. They were then sealed and incubated at 37 °C. After 24 h, the plates were observed for the inhibition of the selected bacteria. The zone of inhibition was measured and expressed in millimetres as an indication of antibacterial activity.

### Morphological identification of endophytic bacteria

Pure bacterial colonies were subjected to Gram staining to investigate their morphological physiognomies such as shape and Gram stain reaction. Briefly, the bacterial isolates were spread on the glass slides and heat fixed using Bunsen burner. The crystal violet was used as a primary stain, followed by iodine solution. It was decolorized by ethanol after washing with tap water. The slides were flooded with safranin and rinsed with tap water. Lastly, the slides were viewed at 1000X magnification using a compound bright- field microscope [31].

### Molecular identification of endophytic bacteria

Five isolates (Isolate 1–5) were further identified using 16S rRNA sequence analysis by Inqaba Biotechnical Industries (Pty) Ltd. Briefly, DNA extraction was done using a ZR Fungal/Bacterial Kit™ according to the manufacturer’s instructions. Polymerase chain reaction (PCR) was run to amplify the 16S rDNA gene of the endophyte with the primers 16S- 27F: 5′AGAGTTTGATCMTGGCTCAG- 3′ and 16S- 1492R: 5′- CGGTTACCTTGTTACGACTT-3′, using DreamTaq™ DNA polymerase. PCR products were gel extracted and sequenced in the forward and reverse directions on the ABI PRISM™ 3500xl Genetic Analyser. The PCR products were cleaned with ExoSAP-it™ following the manufacturer’s recommendations. Purified sequencing products were analysed using CLC Bio Main Workbench v7.6, followed by a BLAST search using the National Center for Biotechnology Information (NCBI) database to identify the closest bacterial species [32].

### Sensitivity assay

Antimicrobial susceptibility assay was performed using the Kirby–Bauer disk diffusion method. Different antimicrobial agent that include; clindamycin 2 μg, ertapenem 10 μg, imipenem 10 μg, kanamycin 30 μg, gentamicin 10 μg, cephalothin 5 μg, amoxicillin 10 μg and meropenem 10 μg were used. *E. coli* (ATCC 25922) and *P. aeruginosa* (ATCC 27853) served as the reference strains alongside the test strain. The zones of inhibition were interpreted in accordance to the Clinical Laboratory Standards Institute (CLSI) guidelines [33].

### Metabolites extraction

The secondary metabolites from Isolate 1 were obtained by inoculating 200 μl of bacterial suspension into 500 ml of NB. The culture was incubated at 28 °C for 5 days on a rotating shaker at 130 rpm. The broth culture was centrifuged at 5000 rpm for 30 min. The supernatant was collected and the pellet discarded. The supernatant was extracted with an equal volume of ethyl acetate (500 ml) and then left overnight at 4 °C. The solvent phase containing the extracted secondary metabolites was separated using separating funnel. The extract was evaporated using a rotary evaporator at 40 °C with 90 rpm to yield the crude metabolites. The residue was re-dissolved in methanol. The concentrated crude extract was then stored at 4 °C for further studies [34].

### Determination of antibacterial activity using crude extract

#### Agar well diffusion

Preliminary antibacterial activity of the crude extract was determined by agar diffusion method as described previously. Ten percent of DMSO was used as a negative control while ciprofloxacin (20 μg/ml) served as a positive control [32].

#### Serial microdilution method

The extract was submitted to further analysis to detect its minimum inhibitory concentration (MIC) using microdilution method [35]. The stock solution of 100 mg/ml of the extract was prepared in 10% dimethyl sulfoxide (DMSO). Micro-serial dilution was carried out using MH broth in a range of 50–0.313 mg/ml. About 100 μl of the fresh selected bacterial suspension at a density of 1 × 10^6^ CFU/min^− 1^, was pipetted into the wells. Ten percent of DMSO was used as a negative control while ciprofloxacin (20 μg/ml) served as a positive control. The plates were sealed and incubated at 37 °C overnight. Afterwards, 40 μl of 0.2 mg/ml of piodonitrotetrazodium violet (INT) solution was added to each well and incubated at 37 °C for 30 min. The MIC was perceived as the lowest concentration of the extract that inhibited the bacterial growth.

### Evaluation of minimum bactericidal concentration (MBC)

MBC was assessed by removing a loopful of each culture from the wells that had no bacterial growth. They were then streaked on different sterile MH agar plates. The agar plates were incubated at 37 °C for 24 h. The lowest concentration of the extract that exhibited the complete killing of the selected bacterial test species was considered as the MBC [36].

### 2, 2-diphenyl-1-picrylhydrazyl (DPPH) radical scavenging assay

The DPPH free radical scavenging activity of the extract was investigated in a sterile 96-well plate. The DPPH (0.02 mg/mL) was mixed (1:1 v/v) with the extract at different concentrations. The mixtures were made to stand at room temperature (25 °C) for 30 min in darkness and the absorbance was read at 517 nm by using microplate reader. The extract without DPPH was used as the blank while ascorbic acid (AA) and butylated hydroxyl anisole (BHA) served as the positive controls. The percent inhibition of DPPH radical was measured by the using the formula: %DPPH scavenging activity = [Ao – A1 / Ao] × 100 where, A1 and Ao equal the absorbance at 517 nm of the control and the test, respectively. The median inhibitory concentration (IC_50_) of the extract against DPPH was calculated graphically [37].

### 2, 2′-azino-bis-3-ethylbenzothiazoline-6-sulfonic acid (ABTS) radical scavenging assay

The ABTS free radical scavenging activity of the extract was evaluated by using serial dilution method. ABTS solution (0.003 g/mL) was mixed (1:1 v/v) with the extract at different concentrations. Each mixture was made to stand for 15 min at 25 °C and the absorbance was read at 734 nm by using microplate reader. The extract without ABTS solution served as blank. Ascorbic acid (AA) and butylated hydroxyl anisole (BHA) were used as the positive controls. The percent inhibition of ABTS radical was calculated by the following formula: %ABTS scavenging activity = [Ao – A1 / Ao] × 100 where, A1 and Ao equal the absorbance recorded at 734 nm of the control and the test, respectively. The IC_50_ of the extract against ABTS was calculated graphically [38].

### Analysis of volatile compounds

The components of the extract were analysed by using gas chromatography-mass spectrometry (GC-MS). The temperature of the GC oven was initially set at 40 °C for 3 min and subsequently increased by 5 °C per minute to 220 °C. The temperature of the injector was programmed to 250 °C, and the flow rate of the helium gas was 1.0 ml per minute, with a 10:1 split ratio. The ion source temperature of the MS system was put at 250 °C with the voltage of 70 eV. The evaluation was performed two times [39].

### Software and statistical analysis

All the experimentations were done in triplicates and the data were subjected to one-way analysis of variance using Graph Pad Prism TM 6.1. Arrow bars represented the standard deviation and values with different alphabets represent the significant difference (*p* < 0.05).

## Results

### Isolation and surface sterilization

The surface of the leaves was sterilised by sequentially immersing them in 70% ethanol, 2% sodium hypochlorite and 10% sodium bicarbonate. This protocol was effective as the control plates did not reveal any bacterial growth after 5 days. Nutrient agar plates inoculated with *A. cordifolia* leaf samples showed morphologically different colonies. The number of CFU per gram fresh weight of the isolates was investigated. The results showed that the leaves had 9.2 × 103 CFU/g^− 1^ (Table 1).

**Table 1:** Endophytic bacterial population from the fresh leaves of *A. cordifolia*

Sample	Un-diluted (CFU/g^−1^)	10^− 1^ (CFU/g^− 1^)	10^− 2^ (CFU/g^− 1^)
Leaves	TN	TN	9.2 × 10^3^

TN denotes too numerous to count

### Enzyme and antimicrobial agent production

The isolates were all screened qualitatively on solid casein, cellulose, tween 80 and starch media for the four industrially important enzymes namely; proteases, cellulase, esterase and amylases. All isolates showed the ability to produce two or more of the tested enzymes. About 38, 62, 38 and 77% of the isolates produced proteinase, amylase, esterase and cellulase, respectively (Table 2). Isolate 1–5 showed the ability to produce all the tested enzymes. The isolates were further screened for antimicrobial agent production using broth fermentation cultures. Isolate 1 did also show broad spectrum against all tested clinical human pathogens (Table 2).

**Table 2:** Enzyme and antimicrobial agent production

Isolates	Enzyme production			Zone of inhibition				
	Proteinase	Amylase	Esterase	Cellulase	***E. coli*** (ATCC 25922)	***P. mirabilis*** (ATCC 25933)	***B. cereus*** (ATCC 10102)	***S. aureus*** (ATCC 25925)
1	+	+	+	+	++	++	+++	+++
2	–	+	+	+	++	–	++	++
3	+	+	–	+	++	–	++	++
4	–	+	+	+	–	++	+	+++
5	+	–	+	+	–	–	+	+++
6	–	+	–	+	–	–	+	+
7	–	+	–	+	+	–	+	–
8	+	–	+	–	+	–	++	++
9	+	+	–	–	+	–	–	+
10	–	–	–	+	–	–	+	+
11	–	+	–	+	–	+	+	+
12	–	+	–	–	–	+	++	+++
13	+	–	+	+	–	–	+	+

+ denotes enzyme production and - denotes no enzyme production. Inhibition zone diameter index: + (≤ 9 mm) weak activity, ++ (10–20 mm) moderate activity, +++ (≥21 mm) strong activity and -denotes no activity

### Identification of the endophytes

The isolates were selected for morphological characterization using Gram staining. Twelve isolates were Gram negative rods while one isolate was a Gram positive rod (Table 3). Molecular characterization of the endophytic bacteria indicate that they are different bacterial species. The final taxonomic status of 5 selected endophytic bacteria isolates was determined by 16S rRNA gene sequencing. The sequence alignment showed the similarity in a range of 97–99% in BLAST with different strains of *Pseudomonas aeruginosa* namely; *P. aeruginosa* CP43328.1, *P. aeruginosa* CP033432.1, *P. aeruginosa* LR657304.1, *P. aeruginosa* CP044006.1 and *P. aeruginosa* CP032126.1 (Table 3).

**Table 3:** Identities and morphological characteristics of the endophytic bacterial isolates

Isolates	Assigned bacterial Names	GenBank accession number	Similarities (%)	Colony morphology	Gram reaction
1	***Pseudomonas aeruginosa***	**CP043328.1**	**99**	**Rod**	**Negative**
2	*Pseudomonas aeruginosa*	CP033432.1	97	Rod	Negative
3	*Pseudomonas aeruginosa*	LR657304.1	97	Rod	Negative
4	*Pseudomonas* *Aeruginosa*	CP044006.1	98	Rod	Negative
5	*Pseudomonas* *aeruginosa*	CP032126.1	98	Rod	Negative
6	*–*	–	–	Rod	Negative
7	*–*	–	–	Rod	Positive
8	*–*	–	–	Rod	Negative
9	*–*	–	–	Rod	Negative
10	*–*	–	–	Rod	Negative
11	*–*	–	–	Rod	Negative
12	*–*	–	–	Rod	Negative
13	*–*	–	–	Rod	Negative

### Susceptibility pattern of *P. aeruginosa* CP43328.1

Based on the outstanding results showed by Isolate 1 (*P. aeruginosa* CP43328.1) during screening methods, Kirby-Bauer method was used to evaluate its susceptibility patterns using different antibiotics. The isolate was resistant to clindamycin, ertapenem, cephalothin, penicillin G, kanamycin and amoxicillin but sensitive to imipenem, meropenem and gentamycin (Table 4).

**Table 4:** Antibiotic susceptibility pattern of *Pseudomonas aeruginosa* CP43328.1; inhibition zones

Isolate	MEM	IML	KF	ETP	DA	K	A	GM
	23 ± 0.0	24 ± 0.0	0	0	0	9 ± 0.0	0	18 ± 1.2

Key: *MEM* meropenem, *IML* imipenem, *KF* cephalothin, *ETP* ertapenem, *DA* clindamycin, *K* kanamycin, *A* amoxicillin and *GM* gentamicin

### Extraction of secondary metabolites

Based on the outstanding results obtained during screening of enzymes and antibiotic production, secondary metabolites from Isolate 1 (*P. aeruginosa* CP043328.1) were extracted. The isolate yielded 0.6 g/500 ml of the extract.

### Antibacterial activity of the extract

The tests for the antibacterial potency of the extract was evaluated against the selected bacterial pathogens using the agar diffusion method and results are demonstrated in Table 5. The extract demonstrated significant inhibitory ability against all selected bacterial strains. It was mostly effective against *S. aureus* (ATCC 25925), with the maximum inhibition zone of 31 ± 1.25 mm.

**Table 5:** Antibacterial activity of the extract evaluated by agar diffusion method

concentration	Zone of inhibition (mm)			
	***E. coli*** (ATCC 25922)	***P. mirabilis*** (ATCC 25933)	***B. cereus*** (ATCC 10102)	***S. aureus*** (ATCC 25925)
4 mg/ml	18 ± 1.25	17 ± 1.00	24 ± 1.41	31 ± 1.25

Microdilution method was used to investigate the minimum inhibition concentration (MIC) of the extract. MIC of the secondary metabolites ranged from 0.098 mg/ml to 0.39 mg/ml. The profound antibacterial activity of the extract was against the Gram-positive bacteria *B. cereus* (ATCC 10102) and *S. aureus* (ATCC 25925) with MIC value of 0.098 mg/ml (Table 6). Although the extract was more pronounced against Gram positive bacterial strains, it also did demonstrate remarkable antibacterial activity against Gram negative bacteria *E. coli* (ATCC 25922) and *P. mirabilis* (ATCC 25933) with the MIC of 0.39 mg/ml. Moreover, ciprofloxacin showed higher MIC value (25 mg/ml) against the tested strains when compared to the extract. Minimum bactericidal concentration (MBC) of the extract was evaluated against the selected bacterial strains. The extract showed only bactericidal effect against *S. aureus* (ATCC 25925) with the MBC of 25 mg/ml (Table 6).

**Table 6:** MIC and MBC values of the extract against the selected bacterial strains

Bacteria	Extract		Ciprofloxacin	
	MIC (mg/ml)	MBC (mg/ml)	MIC (mg/ml)	MBC (mg/ml)
*B. cereus* (ATCC 10102)	0.098 ± 0	> 50 ± 0	25 ± 0	> 50 ± 0
*S. aureus* (ATCC 25925)	0.098 ± 0	25 ± 0	25 ± 0	> 50 ± 0
*E. coli* (ATCC 25922)	0.391 ± 0	> 50 ± 0	25 ± 0	> 50 ± 0
*P. mirabilis* (ATCC 25933)	0.391 ± 0	> 50 ± 0	25 ± 0	> 50 ± 0

### 2, 2-diphenyl-1-picrylhydrazyl (DPPH) radical scavenging assay

**Fig. 1:**
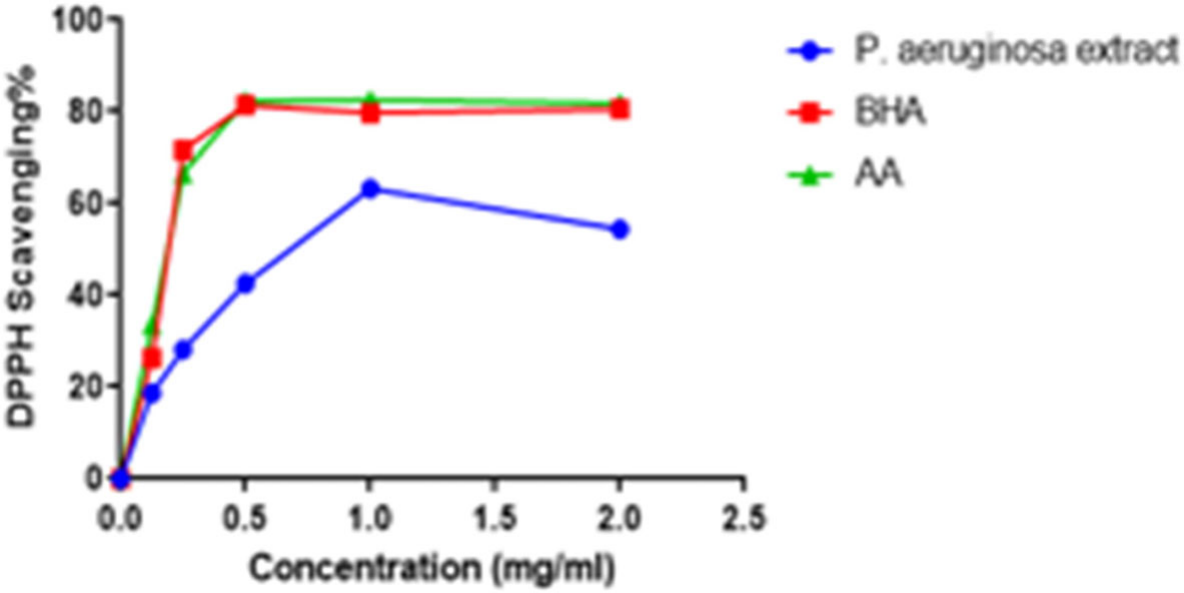
DPPH scavenging activity of the extract, ascorbic acid and BHA

The scavenging activity of the extract against DPPH is shown in Fig. 1. The extract exhibited maximum DPPH scavenging activity of 63% at 1.0 mg/ml. It also demonstrated the IC_50_ value of 0.650 mg/ml, which was higher than that of ascorbic acid (0.200 mg/ml) and BHA (0.188 mg/ml).

### 2, 2′-azino-bis-3-ethylbenzothiazoline-6-sulfonic acid (ABTS) radical scavenging assay

**Fig. 2:**
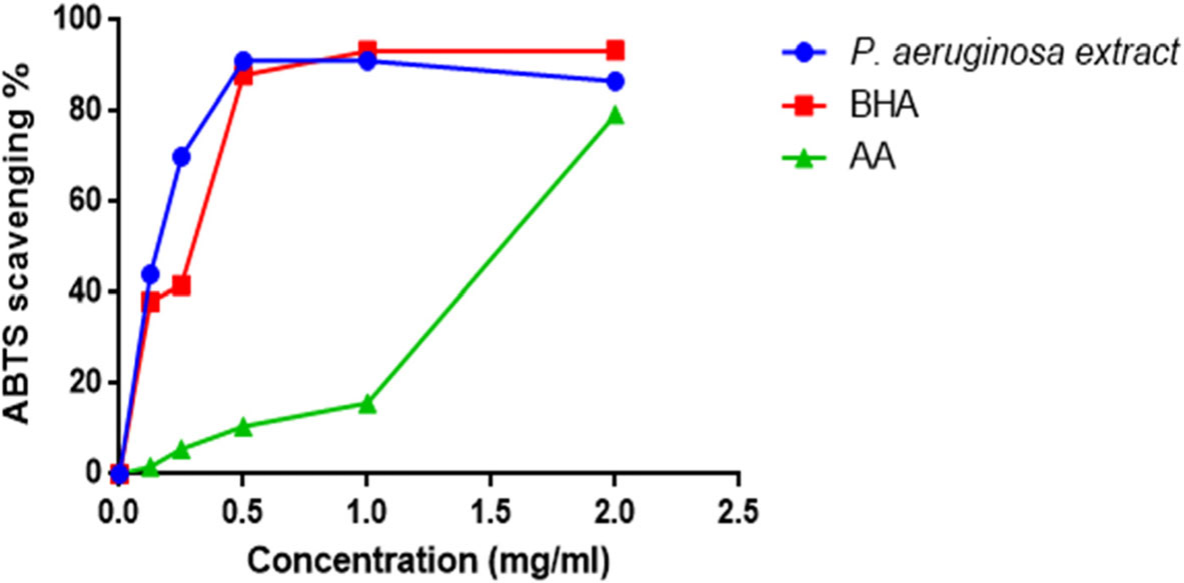
ABTS scavenging activity of the extract, ascorbic acid and BHA

The ABTS radical scavenging activity of the extract is shown in Fig. 2. The scavenging activity of the extract increased up to 91% at 0.5 mg/ml. The extract had potent antioxidant activity with an IC_50_ value of 0.150 mg/ml. Ascorbic acid and BHA had IC_50_ of 0,258 mg/ml and 0,300 mg/ml, respectively.

### Analysis of bioactive compounds

GC-MC chromatogram displayed a total of 15 compounds (Table 7). They are diisooctyl phthalate (50.51%), 9-octadecenamide (10.44%), [1, 2, 4] oxadiazole, 5-benzyl-3- (thiophen-2-yl) (5.70%), 2-dodecenoic acid (5.51%), trans-2-decenoic acid (4.57%), xanthoxylin (3.74%), L-proline, N-valeryl-, undecyl ester (2.86%), L-proline, N-valeryl-, tetradecyl ester (2.61%), pyrrolo [1,2-a]pyrazine-1,4-dione, hexahydro-3-(2-methylpropyl) (2.37%), pentadecanoic acid (2.13%), cis-9-octadecenoic acid (2.13%), cyclohexanone, 4-methyl, O-methyloxime (2.04%), 3-nonynoic acid (1.88%), benzonitrile, 2-(2-pyridinyl) (1.88%) and ergotaman-3′,6′,18-trione,9,10-dihydro-12′-hydroxy-2′-methyl-5′-(phenylmethyl) (1.68%). (TLC) was carried out to investigate chemical compounds in the extract.

**Table 7:** Chemical constituents of the extract

Number of compounds	Compounds	Area (%)
1	Ergotaman-3′,6′,18-trione,9,10-dihydro-12′-hydroxy-2′-methyl-5′-(phenylmethyl)	1.68
2	3-Nonynoic acid	1.88
3	Benzonitrile, 2-(2-pyridinyl)	1.88
4	Cyclohexanone, 4-methyl-, O-methyloxime	2.04
5	Pentadecanoic acid	2.13
6	cis-9-Octadecenoic acid	2.13
7	Pyrrolo[1,2-a]pyrazine-1,4-dione, hexahydro-3-(2-methylpropyl	2.37
8	L-Proline, N-valeryl-, tetradecyl ester	2.61
9	L-Proline, N-valeryl-, undecyl ester	2.82
10	Xanthoxylin	3.74
11	trans-2-Decenoic acid	4.57
12	2-Dodecenoic acid	5.51
13	[1, 2, 4] Oxadiazole, 5-benzyl-3-(thiophen-2-yl)	5.70
14	9-Octadecenamide	10.44
15	Diisooctyl phthalate	50.51

## Discussion

The multi-drug resistance of pathogenic microorganisms is getting more and more severe yearly due to misuse of drugs [40]. Endophytes, especially those from medicinal plants are reported to be prolific producers of variety of metabolites with profound bioactivities. Moreover, their metabolites are biodegradable and easy to be produced on a large scale [41]. Thus, bacterial endophytes from *A. cordifolia* CIX1 were isolated for production of bioactive compounds.

A variety of chemical disinfectants have been employed for surface sterilization of excised plant tissues to remove epiphytes. However, immersion of tissue in ethanol, sodium hypochloride and bicarbonate have shown significant success in different studies [10]. The effectiveness of surface sterilization of the leaves from *A. cordifolia* CIX1 was confirmed as there was no microbial growth observed after 5 days. This indicate that the surface sterility method was effective at inhibiting growth of epiphytic bacteria and fungi. Thus, the subsequence isolates can be considered as true endophytic bacteria. The leaves had CFU value of 9.2 × 103 (Table 1). The diversity of isolated endophytic bacteria dependent on the media used during isolation [42]. Our results demonstrated that NA did support the growth of endophytic bacteria. Thirteen endophytic strains were isolated from the fresh leaves of *A. cordifolia* CIX1. It can therefore be assumed that the ecological success of *A. cordifolia* CIX1 in the north part of Kwazulu-Natal could not only be associated to the conducive climate and fertile soil but also to its association with active endophytes as they are known to have symbiotic relationship.

Currently, microorganisms are attracting an increasing attention as source of for new enzymes because enzymes derived from microbes are relatively stable and active than corresponding enzymes derived from plants or animals [43]. In the study, the qualitative analysis of enzyme production namely; protease, cellulase, esterase and amylase was done. Most of the isolate tested positive for enzyme production. Our observations are in agreement with previous reports that illustrated endophytes such as *Pseudomonas* sp. as producers of extracellular amylase, esterase, cellulose and proteinase [44]. The hydrolytic enzymes are perceived to play an important role for the colonization of *A. cordifolia* CIX1 by the isolated endophytes.

All the morphologically identified bacterial endophytes were of *Pseudomonas* sp. The isolate of interest (Isolate 1) exhibited 99% similarity with *Pseudomonas aureuginosa* CP043328.1. Most studies have reported that *Pseudomonas* sp. are one of the main endophytic bacteria often found associated with most of the medicinal plants. Thus, our study affirmed these reports. Endophytic *Pseudomonas* sp. have been used as biocontrols against phytopathogens [45].

Antimicrobial resistant genes, in addition to clinical pathogens, are also present in environmental isolates. Isolate-*P. aureuginosa* CP043328.1 was sensitive to meropenem, imipenem and gentamycin (Table 2). This suggest that these compounds (antibiotics) are not present as secondary metabolites in *P. aureuginosa* CP043328.1. Moreover, these antibiotics can be used if there is an outbreak due to *P. aureuginosa* CP043328.1. Ethyl acetate solvent was used for extraction of the secondary metabolites and the yield was 0.6 g/500 ml. Ethyl acetate is the good solvent for extraction of active metabolites from bacterial endophytes as it has medium polarity (dissolve both polar and non- polar active compounds) [46]. In addition to medium polarity, the solvent extract low and high molecular weight polyphenols.

In therapeutic applications, secondary metabolites are significant as they often demonstrate among others, efficacy against multidrug-resistant bacteria. Thus, secondary metabolites from Isolate 1-*P. aeruginosa* CP043328.1 were extracted and antibacterial potency of the extract was evaluated against the selected bacteria. The extract showed a broad-spectrum activity against all tested bacterial strains (Table 6). It was more pronounced against Gram positive bacterial strains. Gram-negative bacteria, in addition to a thin peptidoglycan layer (2 to 7 nm), do possess approximately 7 to 8 nm of the outer membrane. This outer membrane consists of an additional protective lipopolysaccharide layer that exhibits toxicity and antigenicity against antibacterial agents [35]. It was therefore concluded that the resistance shown by the Gram-negative bacteria as compared to Gram-positive bacteria to the extract was as the result of the mechanism of action of this layer. Gram positive bacteria do not possess this layer and therefore they were highly sensitive to the extract. Gram positive bacteria allowed the direct contact of the extract constituents with the phospholipid bilayer of the cell membrane, enabling the extract to inhibit bacterial growth easily. Microbial and plant extracts that have noteworthy antibacterial activities are those with MIC values less than 1 mg/ml [47]. Thus, the profound activity of the extract suggests the potential use as a therapeutic agent since it demonstrated MIC values in a range of 0.098–0.391 mg/ml against the selected pathogens. Moreover, it is interesting to note that ciprofloxacin had higher MIC value (25 mg/ml) against the tested strains when compared to the extract. This implied that that the bacterial strains had developed ciprofloxacin resistance and affirms the need for novel antibacterial agents. Furthermore, the extract exhibited bactericidal effect against *S. aureus* (ATCC 25925) with the MBC of 25 mg/ml.

Compounds with scavenging activities against free radicals have gained constant attention in recent years. These compounds have potential to nullify disease progression caused by excess free radicals [48, 49]. Since there are few studies investigating the antioxidant activity of extracts from endophytic microorganisms, the evaluation of antioxidant activity of the extract was imperial. The extract showed poor activity against DPPH radical (Fig. 1) and moderate activity against ABTS radical (Fig. 2). Molyneux, 2004 classified the antioxidant activity where the highly active compounds have IC_50_ values < 0.05 mg/ml, the active category have IC_50_ values of 0.05–0.1 mg/ml, medium category have IC_50_ values of 0.1–0.15 mg/ml, and weak categories have IC_50_ values of 0.151–0.2 mg/ml. Thus, the results clearly show that the extract has promising ABTS scavenging activity and could be a potential source of natural antioxidant.

*Pseudomonas aeruginosa* is known for producing a wide variety of metabolites with antimicrobial and antioxidant effects. The identified volatile compounds, xanthoxylin, trans-2-decenoic acid, [1, 2, 4] oxadiazole, 5-benzyl-(thiophen-2-yl), dodecenoic acid, 3-nonynoic, pyrrolo [1,2-a]pyrazine-1,4-dione, hexahydro-3-(2-methylpropyl), pentadecanoic acid and diisooctyl phthalate are recognised for possession of antimicrobial activity [50–57]. cis-9-Octadecenoic acid, 2-dodecenoic acid and 9-octadecenamide are reported to possess antioxidant property [58, 59]. It was therefore assumed that the profound antibacterial and antioxidant activity of extract was due to synergistical activities of the identified bioactive compounds.

## Conclusions

Out of 13 isolates, Isolate 1 (*P. aeruginosa* CP43328.1) produced all the tested enzymes and showed the ability to produce antimicrobials that were effective against all the tested pathogens. Its secondary metabolites demonstrated antibacterial activity with MIC values ranging from 0.098–0.391 mg/ml. It had bactericidal effect against *S. aureus* (ATCC 25925) at 25 mg/ml. It also illustrated potent ABTS scavenging activity with an IC_50_ value of 0.051 mg/ml. The extract showed a total of 15 volatile compounds with diisooctyl phthalate (50.51%) and [1, 2, 4] oxadiazole, 5-benzyl-3 (10.44%) being the major compounds. *P. aeruginosa* CP43328.1 has a potential to serve as a source of pharmacologically important compounds. Further studies, quantitative analysis of the detected enzymes and evaluation of the mechanisms involved in antibacterial activity are imperial.

## Data Availability

The datasets used and/or analysed during the current study available from the corresponding author on reasonable request.

## Abbreviations

NA: nutrient agar
MH: Mueller Hinton
CFU: colony forming units
TLC: thin layer chromatography
NCBI: National Center for Biotechnology Information
MIC: minimum inhibitory concentration
MBC: minimum bactericidal concentration
ATCC: American Type Culture Collection
DMSO: dimethyl sulfoxide
INT: piodonitrotetrazodium violet
IC_50_: half maximal inhibitory concentration
ABTS: 2, 2′-azinobis (3-ethylbenzothiazoline-6-sulfonic acid)
BHA: butylated hydroxyl anisole
MEM: meropenem
IML: imipenem
OB: cloxacillin
KF: cephalothin
ETP: ertapenem
DA: clindamycin
CLSI: Clinical Laboratory Standards Institute
BLAST: basic local alignment search tool
